# VIROME: a standard operating procedure for analysis of viral metagenome sequences

**DOI:** 10.4056/sigs.2945050

**Published:** 2012-07-27

**Authors:** K. Eric Wommack, Jaysheel Bhavsar, Shawn W. Polson, Jing Chen, Michael Dumas, Sharath Srinivasiah, Megan Furman, Sanchita Jamindar, Daniel J. Nasko

**Affiliations:** 1 Delaware Biotechnology Institute, University of Delaware, Newark, DE 19711; 2 Institute for Genome Sciences, University of Maryland School of Medicine, Baltimore, MD 21201; 3 California Institute for Telecommunications and Information Technology (Calit2), University of California San Diego, San Diego, CA 92093

**Keywords:** environmental sequencing, shotgun metagenomics, viral ecology, ORFan

## Abstract

One consistent finding among studies using shotgun metagenomics to analyze whole viral communities is that most viral sequences show no significant homology to known sequences. Thus, bioinformatic analyses based on sequence collections such as GenBank nr, which are largely comprised of sequences from known organisms, tend to ignore a majority of sequences within most shotgun viral metagenome libraries. Here we describe a bioinformatic pipeline, the Viral Informatics Resource for Metagenome Exploration (VIROME), that emphasizes the classification of viral metagenome sequences (predicted open-reading frames) based on homology search results against both known and environmental sequences. Functional and taxonomic information is derived from five annotated sequence databases which are linked to the UniRef 100 database. Environmental classifications are obtained from hits against a custom database, MetaGenomes On-Line, which contains 49 million predicted environmental peptides. Each predicted viral metagenomic ORF run through the VIROME pipeline is placed into one of seven ORF classes, thus, every sequence receives a meaningful annotation. Additionally, the pipeline includes quality control measures to remove contaminating and poor quality sequence and assesses the potential amount of cellular DNA contamination in a viral metagenome library by screening for rRNA genes. Access to the VIROME pipeline and analysis results are provided through a web-application interface that is dynamically linked to a relational back-end database. The VIROME web-application interface is designed to allow users flexibility in retrieving sequences (reads, ORFs, predicted peptides) and search results for focused secondary analyses.

## Introduction

Scientific appreciation of the true extent of microbial diversity and the composition of natural microbial communities now firmly rests on two approaches which utilize environmental DNA sequence data: 1) molecular phylogenetic analysis of single genes which are broadly shared among microbial groups (e.g., the 16S rRNA gene); and 2) shotgun metagenomic sequencing of environmental DNA. Oftentimes, the principal objective of marker gene studies is to utilize molecular phylogenetic analyses to make inferences about the taxonomic diversity of microorganisms within an environment [1] or the composition (i.e., richness and evenness) of entire microbial communities [2]. Overwhelmingly, the 16S rRNA gene, which is omnipresent among cellular life, has been used for these studies. One shortcoming of 16S gene studies is that in many cases connections between 16S molecular phylogeny and the physiological capabilities of a microorganisms are unknown or tenuous [3-5]. In an effort to address this shortcoming, investigators have increasingly turned to shotgun sequencing of environmental DNA as a means to assess the potential physiological capabilities of microorganisms within natural communities [6-8]. While shotgun metagenomic studies have revealed new insights on possible physiological diversity within natural communities of prokaryotes, these data are typically limited to those populations at highest abundance. The genome size of most prokaryotes (~1.5 to 2.5 Mb) means that extraordinary sequencing effort is required to obtain data on the genetic composition of minority populations using shotgun metagenomic approaches [9]. For eukaryotic microorganisms, the issue of genome size is particularly acute and has prevented attempts at shotgun metagenomic characterization of these microorganisms. Ironically, while we are increasingly aware of the taxonomic breadth of microbial diversity according to small subunit rRNA molecular phylogeny, we know little of the genetic capabilities of many microbial phyla. The disconnect between taxonomy and function has been a driving rationale behind microbial genome sequencing efforts such as the *Genomic Encyclopedia of Bacteria and Archaea* [10,11].

In the case of viruses, the lack of a single, universally shared and phylogenetically informative gene has limited the ability of researchers to easily assess the diversity and composition of natural viral assemblages [9,12]. However, in contrast to prokaryotes and eukaryotes, the small genome sizes of most environmental viruses (~50 to 100 kb) means that it is possible to obtain genetic sequence data from a broad cross-section of viral populations using modest levels of shotgun DNA sequencing. Thus, shotgun metagenome data has provided a means to both estimate the diversity and composition of viral communities [13,14] and assess the potential genetic capabilities of natural viral populations [15]. Indeed, shotgun metagenomics may find its best application in ecological studies of viruses. While shotgun metagenomics promises to unlock the black-box of viral diversity, in practice, both viral genome and metagenome sequence data have proven intractable for gene annotation pipelines designed for microbial sequence data. Investigators routinely report that a after exhaustive homology search analysis, half or more of the genes identified within a viral genome or metagenome are unknown (i.e., homologous to a hypothetical or uncharacterized protein) or novel (i.e., ORFans with no significant homology match) [12,16]. To address this shortcoming, boutique databases and bioinformatic tools have been developed to assist with characterizing viral genes. Here we report on a bioinformatics pipeline, the Viral Informatics Resource for Metagenome Exploration (VIROME) which has been designed to classify all putative ORFs from viral metagenome shotgun libraries and thus provide a means of exhaustively characterizing viral communities.

## Requirements

The VIROME analysis pipeline relies on three subject protein sequence databases, five annotated databases, the UniVec database, and CD-Hit 454 [17]. The UniVec database is used to screen reads for the presence of contaminating vector sequences within metagenome sequence reads [18]. The CD-Hit 454 algorithm is used to screen sequence libraries from the 454 pyrosequencer for the presence of false duplicate sequences known to arise from the 454 library construction protocol [17]. A taxonomically diverse collection of ~30,000 ribosomal RNA genes (5S, 16S, 18S, and 23S) is used to detect the presence of ribosomal RNA homologs within sequence libraries. The UniRef 100 peptide database contains clusters of identical peptides (>11) within the UniProt knowledgebase and is used to detect viral metagenome sequences with similarity to known proteins [19,20]. Connections between UniRef sequences and five annotated protein databases (SEED [21] ; ACLAME [22]; COG [23]; GO [24] and KEGG [25) are maintained within a relational database which allows for display of multiple lines of evidence from a single BLASTP homology result.

The MetaGenomes On-line (MGOL) peptide database contains nearly 49 million predicted peptide sequences from 137 metagenome libraries and is used to detect similarity to unknown environmental sequences. Within MGOL, nine libraries are described as ‘Eukaryotic’ since they were obtained from cells > 1 µm in size. Thirty-eight are described as ‘Viral’ (i.e., particles < 0.022 µm) and 89 are described as ‘Microbial’ (i.e., cells between 0.22 and 1 µm in size. One library is described as ‘Microbial/Eukaryotic’ since it was collected from a 0.22 to 5 µm size fraction. With the exception of some of the viral libraries, all MGOL peptides are contained in the CAMERA database [26]. All peptides within the MGOL database were predicted from shotgun metagenome sequences obtained using the Sanger dideoxy chain-terminator sequencing method [27].

## Procedure

**Figure 1:**
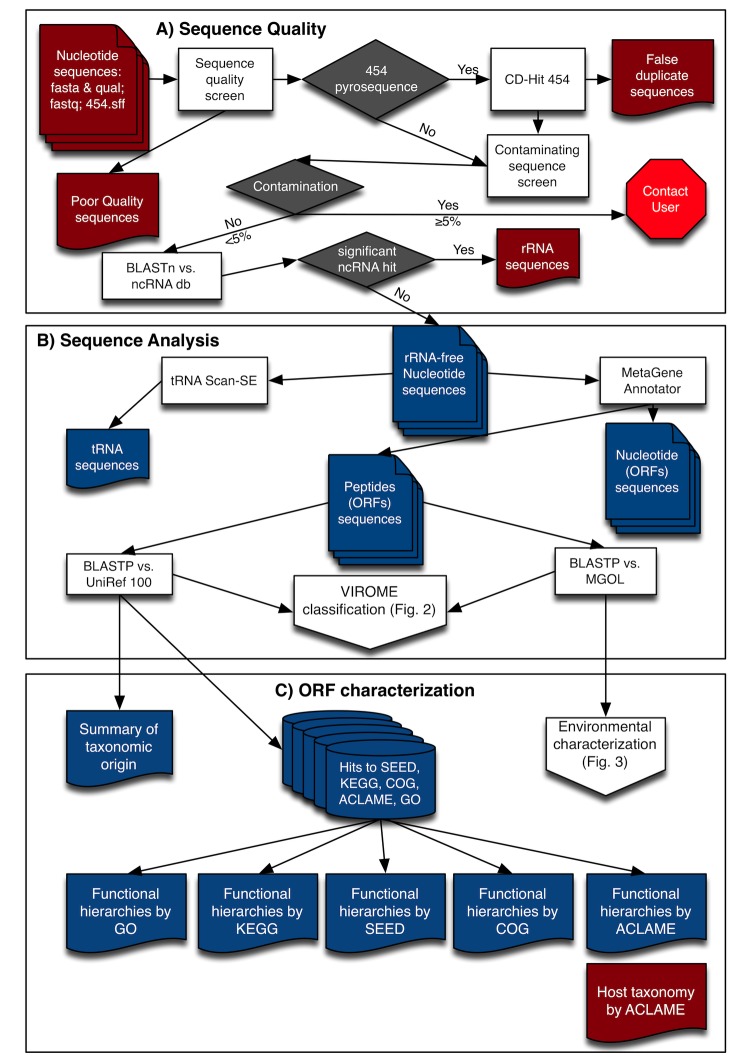
Overview flow-chart of VIROME bioinformatic pipeline. A) Initial screening steps to remove poor quality sequences, false duplicate sequences created during 454 em-PCR library preparation, and rRNA-containing sequences. Contaminating sequence screens includes searches against the UniVec database for vector, linker, and adapter sequences. B) Analysis steps including the identification of tRNA-containing sequences and BLASTP of metagenome peptides against the UniRef 100 and MGOL sequence databases. Significant BLASTP hits have an expectation score of E <0.001. C) Viral metagenome peptide sequences with a significant hit a UniRef 100 sequences are characterized by the taxonomic origin of the homolog and functional information contained within UniRef or the annotated databases. Those metagenome peptides with hits to the MGOL database are characterized according to the environmental origin of their MGOL homologs (Figure 3). Sequences within blue objects are accessible through VIROM web-application interface for viewing or download. Parameters for sequence analyses (rectangles) are given in Table 1.

**Figure 2:**
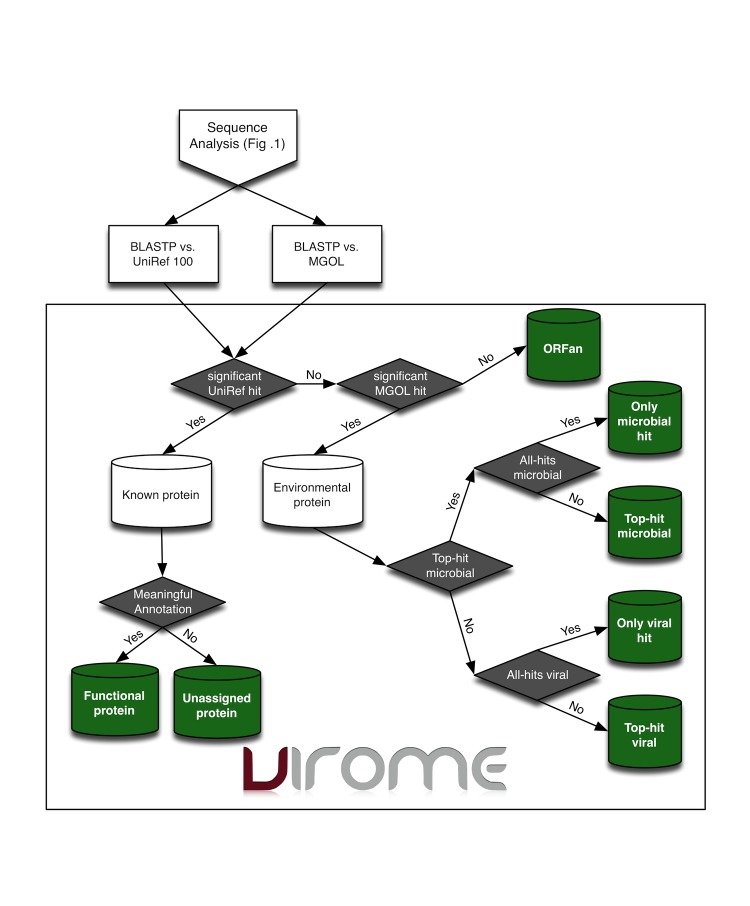
Overview flow-chart of the VIROM classification scheme for environmental peptides. BLAST homology data from the sequence analysis pipeline (Figure 1) serves as input to the classification decision tree. Peptides having a significant hit (E ≤ 0.001) to a sequence in UNIREF 100 are placed in the ‘Known protein’ bin. If one of the homologs has a meaningful annotation, the viral metagenome predicted peptide is considered a ‘Functional protein’. If not, the peptide is considered an ‘Unassigned protein’. Peptides having only a significant hit to an environment peptide in the MGOL database are placed in the ‘Environment protein’ bin. Within this bin, peptides that hit only environmental proteins within either microbial or viral metagenome libraries are classified as ‘Only microbial hit’ or ‘Only viral hit’, respectively. Peptides having hits to protein within viral and microbial metagenome libraries are classified as either ‘Top-hit microbial’ or ‘Top-hit viral’ depending on whether the top BLAST hit came from a microbial or viral metagenome library, respectively. A predicted viral metagenome peptide having no significant hit to a protein within the UniRef 100 or MGOL sequence databases is classified as an ‘ORFan’.

**Figure 3:**
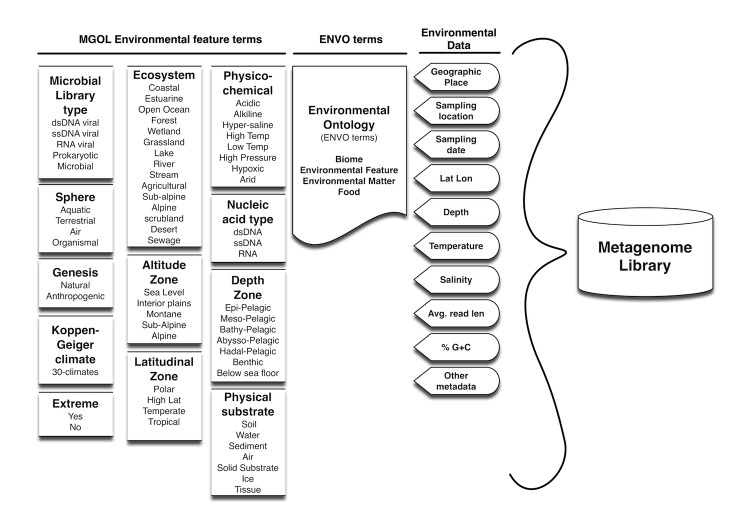
Environmental terms and metadata appended to each library within the MetaGenomes On-Line (MGOL) database. Using the annotation scheme presented in Figure 2, the distribution of significant BLAST hits (E<0.001) to MGOL sequences can be described according to environmental feature terms or ENVO terms.

The VIROME bioinformatics pipeline consists of two consecutive steps: 1) sequence quality screening; and 2) sequence analysis (Figure 1); followed by three parallel steps: 1) functional and taxonomic ORF characterization (Figure 1) [2]; ) ORF classification (Figure 2 and Figure 3) and environmental characterization (Figure 3). A nucleotide sequence file in either fasta & qual, fastq, or the 454 sequencing .sff format is the singular input to the VIROME pipeline. Subsequently, each sequence within the file is trimmed for quality and trimmed of contaminating linker, adapter, and bar-code sequences (Figure 1A). In the case of pyrosequencing data, the native 454 pyrosequencer output (i.e., a .sff file) can be used as an input file. In addition to the screens for contaminating sequence (e.g., vector, linker, or adapter sequences used in the sequencing procedure), 454 sequence libraries are also screened for the presence of false duplicate reads using CD-Hit 454 [17]. After these initial screening steps, nucleotide sequences are scanned for the presence of ribosomal RNA genes using BLASTN against a rRNA subject database. Sequence reads showing significant homology to a rRNA sequence (E ≤ 10^-75^ for a match length of ≥ 150 bp) are removed from the sequence library and a rRNA-free sequence file is generated. Sequences within this new file are scanned for the presence of tRNAs using tRNAscan-SE [28] and open reading frames (ORFs) are predicted using MetaGene Annotator [29] (Fig. 1B). Subsequently, a multi-fasta file of peptide sequences is constructed from the predicted ORFs. The pipeline is flexible enough to also directly utilize a multi-fasta file of peptide sequences; however, with a loss of the rRNA scan and tRNA scan steps. Each peptide within this file is analyzed using BLASTP against the UniRef 100 and MGOL databases.

**Table 1:** Algorithms, parameters, and databases used in the VIROM bioinformatics pipeline

Process	Tool	Parameters	Subject database
Screening of rRNAs	BLASTALL	-p blastn –e 1e-3 –f T – b 1 –v 1 –M BLOSUM62	
Identification of tRNAs	tRNA scan SE	-b G	
ORF calling	MetaGene Annotator	-m	
Known protein identification	BLASTALL	-p blastp –e 1e-1 –F T – b 50 –v 50 –M BLOSUM 62	UNIREF 100
Environmental Protein Identification	BLASTALL	-p blastp –e 1e-1 –F T –b 50 –v 50 –M BLOSUM62	MGOL

Predicted viral metagenome peptides having a significant hit to a UniRef 100 protein can be characterized according to the taxonomic origin of the top UniRef 100 BLAST hit (Figure 1C). For those instances where the UniRef homolog also occurs in one of the annotated sequence databases, predicted peptides can be characterized using the functional hierarchical descriptions provided in the GO, ACLAME, KEGG, COG, or SEED databases. The VIROME web-application interface enables users to summarize entire libraries of predicted peptides according to functional hierarchies and subsequently download these summary views as a tab-delimited search result or as a FASTA formatted file of peptides, nucleotide, or read sequences. Additionally, for viral metagenome peptides having a hit against a UniRef protein, the sequence descriptions and BLAST statistics for the top UniRef hit can be displayed in a delimited search view. These top BLAST hit UniRef sequence descriptions are fully searchable with search results appearing in the search view window of the VIROME web-application interface. The flexibility of the VIROME web-application allows for any predicted peptide BLAST results appearing in the search view window to be downloaded as a tab-delimited file of search results or as a FASTA formatted file of peptides, nucleotide, or read sequences.

Because viral peptides with a significant hit to a known protein in the UniRef 100 database typically comprise less than a third of all ORFs in a viral metagenome library [12], an ORF classification scheme was devised to aid investigators in characterizing the genetic diversity of entire viral communities using all predicted peptides within a library. Based on the outcome of BLASTP analyses, each predicted viral metagenome peptide is classified into one of seven VIROME classes (Fig 2). Those predicted peptides showing significant homology (E ≤ 0.001) to a known protein within the UniRef 100 subject database are classified as either a ‘Functional protein’ or an ‘Unassigned protein’ (Figure 2). Viral peptides within the functional protein class have at least one protein homolog that fulfills one or more of the following criteria: has a GO annotation; belongs to a SEED sub-system; has a KEGG Orthology; has a MEGO annotation; or belongs to a cluster of orthologous groups. For ‘Unassigned proteins’ the UniRef homolog of a viral metagenomic peptide may have an association with a sequence in one of the annotated databases, however, there was no meaningful information associated with the sequence. For example, if the SEED entry for a homolog of an unassigned protein has not been assigned to a sub-system, the homolog would be considered as having no meaningful annotation. Because this classification system relies on a stringent criterion (i.e., annotation within the GO, KEGG, SEED, COG or ACLAME databases), it is possible that a small fraction of viral metagenomic peptides within the unassigned protein class have homology to UniRef proteins with an informative sequence description. However, annotation of query sequences by text parsing of homolog sequence descriptions can be notoriously inaccurate; a fact which has driven the development of controlled vocabularies such as the Gene Ontology [24] and prompted our decision to place this restriction on the classification scheme.

Viral metagenomic predicted peptides showing significant homology to only environmental peptides within the MGOL subject database are considered environmental proteins (Figure 2). Because each MGOL sequence is identified as coming from either a viral, microbial, or microbial/eukaryotic metagenome, it is possible to add four additional classifications to environmental proteins. Those predicted peptides having hits to only peptides from viral metagenome libraries are classified as ‘Viral only’. If the top MGOL hit was from a viral library, but the predicted viral metagenome protein also showed homology to a protein within a microbial metagenome library, the environmental protein is classified as ‘Top viral hit’. In a similar way, but with reference to microbial metagenome libraries, predicted peptides within the environmental protein bin are classified as ‘Microbial Only’ or ‘Top microbial hit’. Predicted viral metagenome peptides showing no homology to a protein within the UniRef 100 or MGOL subject databases are classified as ‘ORFans’.

**Figure 4:**
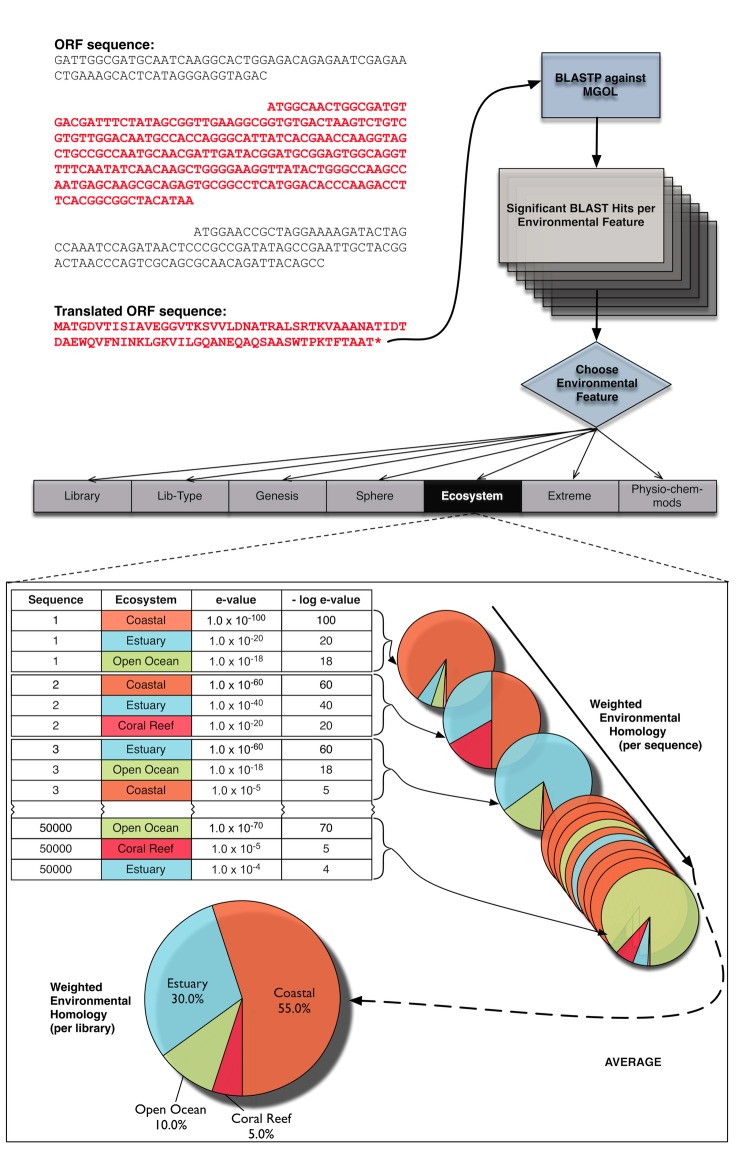
Flow-chart of VIROME environmental annotation. For each predicted viral metagenome ORF, E-scores (E<0.01) of top-hits against each unique library in the MGOL database are summed. Ratios of E-score distribution for each unique MGOL library are calculated. These ratios can be used to examine the prevalence of sequence homologs according to the environmental features of MGOL libraries (e.g., ecosystem, biome, physico-chemical parameters).

Identifying the frequency of particular functional groups of genes within viral metagenome libraries is made possible by the annotated functional information associated with UniRef 100 sequences. In contrast, analyzing subgroups of viral metagenome peptides having homology to only other environmental proteins using environmental or biological criteria was not possible using available sequence databases. Thus, an important goal in developing the MGOL database was the addition of environmental annotation data to each sequence within the database to provide a means for finer levels of classification for viral metagenome peptides (Figure 3). Each metagenome library within MGOL was annotated with common-language terms describing a number of environmental features associated with the original sample from which each metagenomic library was derived. These annotations enable the creation of informative sequence descriptions for each environmental peptide within MGOL. The sequence descriptions contain information about metagenome type, ecosystem, geographic location, and a short descriptive name of the metagenome library [example: Viral metagenome from Agricultural Soil near Delaware Agricultural Experiment Station, Newark, DE, United States (library: MATAPEAKE)]. In addition, Environmental Ontology (Env-O) terms and any available quantitative data such as pH, salinity, temperature, and geospatial coordinates were also included in the annotation of MGOL libraries. Using the environmental feature annotations of MGOL sequences and the VIROME informatics pipeline, it is possible to group viral metagenome peptides according to significant BLAST hits against MGOL peptides. The MGOL environmental feature annotations used for grouping viral metagenome peptides are MGOL library_id, library type, genesis, sphere, ecosystem, or extreme environment and its physio-chemical characteristics (Figure 4). The VIROME web-application provides summaries of MGOL BLAST hit data for viral metagenome peptides according to each of these environmental features using a weighted scheme.

This process is illustrated for the ‘Ecosystem’ environmental feature (Figure 4). For each viral metagenome peptide, all significant MGOL BLAST hits are considered (E ≤ 0.001) and the -log E-scores of the top hits against each unique ecosystem are summed. Subsequently, the ratio of the top hit -log E-score for each individual unique ecosystem to the sum -log E score across all top ecosystem hits is calculated thus providing a weighting of the BLAST homology across ecosystems. The ecosystem having the lowest E-score BLAST hit (i.e., the highest quality hit) would have the largest share of the ecosystem environmental feature characterization for an individual viral metagenome peptide. Subsequently, the weighted analysis of each peptide having similarity to a MGOL sequence(s) can be summed and used to characterize an entire library by a given environmental feature. Because this weighted scoring system considers all significant MGOL hits and not just top BLAST hits, it provides a robust picture of the proportions of viral genetic diversity that are specific to a given environmental context or more broadly shared across contexts. It is often true that the largest weighted frequency for a given MGOL environmental feature is similar to that of the query library. For instance, a query viral library from the Chesapeake Bay, which itself would be defined as an ‘Estuary’ ecosystem, would show ‘Estuary’ as the largest weighted fraction of MGOL hits according to the ‘Ecosystem’ environmental feature. This common observation indicates that many viral genes show specificity to a particular environmental context, supporting reports from previous viral metagenomic studies [30,31].

## Implementation

The sequence quality (Figure 1A) and sequence analysis (Figure 1B) components of the VIROME bioinformatics pipeline are run using a workflow management system called Ergatis [32]. Ergatis has direct access to the executable component scripts and algorithms that comprise the pipeline and can execute computation locally or on a computational grid running Sun Grid Engine. Data from the sequence processing and BLAST analysis components are stored in a MySQL database. Subsequent analyses of these data, which assign viral metagenome peptides to VIROME categories and summarize the distribution of these peptides according to functional or environmental criteria, are done using the MySQL database and custom scripts. These analyses are all conducted within the routine VIROME bioinformatics pipeline and managed with Ergatis. The VIROME web-application was developed using Adobe Flex and runs on any web-browser that supports the Flash plug-in. This architecture ensures that the VIROME web-application is platform and browser independent. Communication between the back-end MySQL database and the VIROME web-application is handled by an Adobe ColdFusion server.

## Discussion

The one consistent finding among viral metagenomics studies has been the high proportion of sequences having no significant homology to a known sequence within one of the large sequence databases (e.g., GenBank, UniRef etc.). Those viral metagenome libraries having the highest frequency of hits to known sequences typically come from marine environments where the hit frequency for longer Sanger reads is around 30% (at a BLAST e-score of ≤0.001) [12]. Sanger libraries from soils show even lower hit rates at ~20%. The lack of homology to known sequences is only exacerbated by the shorter read lengths of next-generation sequencing technology [33] where libraries sequenced using the longest average read length next generation sequencing technology (i.e., 450 bp for the 454 pyrosequencing Ti FLX chemistry) yield hit rates to known sequence databases of less than 20%. In contrast, microbial shotgun metagenome libraries analyzed using the same databases and approaches will yield hit frequencies of ca. 80% [33].

These trends indicate that most viral genes are not represented within the major sequence databases. Viral metagenome hit frequencies are even lower when considering smaller, better annotated databases such as SEED and KEGG. As a result, most metagenomic analyses of the genetic and taxonomic composition of viral communities have been based on small sub-populations of sequences within viral shotgun libraries. This subset of sequences has alerted researchers to the ubiquitous presence of metabolic genes within viruses, genes once thought to exist only in cellular life [34,35], and supported the development of approaches such as MaxiΦ for examining the taxonomic diversity of viral communities [36]. However, relying solely on known sequence homologs ultimately stymies the discovery of novel viral genes that likely encode unique and important biological features of viruses found in nature. Thus, a key motivation behind developing the VIROME pipeline and ORF classification scheme has been to add some level of information to the majority viral metagenome ORFs having no significant hit to a known sequence. This objective has been accomplished through BLAST analysis of predicted viral metagenome ORFs against the ~ 49 million peptides within the Metagenomes On-Line database.

To our knowledge, the only other metagenomics analysis pipeline to include analysis against environmental peptides is the Viral Metagenome Annotation Pipeline (VMGAP) [37]. In the VMGAP pipeline, BLAST analysis against the GenBank env-nr, env-nt databases and a Sanger viral metagenome peptide database are used as three of 13 different annotation evidence types for characterizing a viral metagenome sequence. Predicted proteins having a significant hit to only an environmental sequence are then described as ‘hypothetical protein’, a minimally useful annotation. In contrast, because of the rich metadata accompanying each sequence within MGOL (Figure 3), for each predicted viral metagenome ORF run through the VIROME pipeline it is possible to extract additional biological meaning such as the predominant ecosystems where the peptide occurs and whether the peptide is found only in viruses. In both VMGAP and VIROME, the inclusion of BLAST analysis against environmental peptides improves the informative sequence content of viral metagenomes as compared to analysis using the MetaVir pipeline, which is based solely on homology searches against known viral genome sequences within the NCBI RefSeq database [38]. Through the VIROME pipeline, typically 70% of Sanger read length viral metagenome sequences from aquatic environments obtain a classification other than ORFan.

Another strength of the VIROME analysis pipeline and web-application interface is the ability to retrieve read sequences, predicted ORFs, predicted peptides and top-hit BLAST results according to a large variety of search criteria. This functionality allows for a broad range of sequence retrieval, from individual sequences to whole libraries. For the researcher, the capability of customized sequence retrieval empowers subsequent sequence-based analyses, especially molecular phylogenetic analyses, which are a cornerstone of molecular ecological studies. In addition to customized searches, the VIROME web-application provides a summary display of BLASTP results organized by criteria such as the taxonomic origin of sequence homologs or functional terms associated with sequence homologs from databases such as KEGG, COG, GO, ACLAME, and SEED. Because VIROME links the sequence information from five annotated databases with UniRef 100 sequences, it is possible to garner a great deal of functional information for those sequences hitting known sequences within UniRef 100. MG-RAST uses a similar strategy with the M5NR non-redundant protein database.

In the VIROME web-application interface, views summarizing homology search results according to functional and taxonomic criteria are displayed using fully interactive charts (e.g., pie charts and bar charts) that are dynamically linked to BLAST data. These summaries provide a ready means for researchers to effectively bin sequences according to a variety of criteria for subsequent analyses such as assembly and clustering. Finally, an important practical concern is that the VIROME pipeline is administered and maintained as a web resource and does not require researchers to have access to advance computing infrastructure (e.g., a database server and computational grid). Other metagenomic pipelines such as VMGAP and MetaRep are not available as web-accessible resources and instead require investigators to install and administer the software on local computers, a task requiring significant UNIX systems administration experience [37,39]. Submission of sequence data for VIROME analysis is initiated through the web-application interface. After filling out a simple form, the user is contacted by electronic mail correspondence with further instructions regarding sequence data transfer.

Areas of future development for the VIROME pipeline include the incorporation a sequence assembly component and build search infrastructure to support comparative analyses. Assembly will serve to reduce redundancy in homology search data for a given library, lessen the computational demands of the pipeline, and will be essential for the analysis of Illumina sequence libraries. Currently, no web-accessible metagenomics pipeline includes an assembly step as a routine component of its analysis. With regards to comparative analyses, VIROME output in the form of chart graphics, tab-delimited summary data, and tab-delimited search data can be downloaded and used as input data for qualitative and quantitative comparisons (e.g., multivariate analyses) using third party tools. At present, MetaVir [38] is the only dedicated web-accessible viral metagenomics analysis pipeline which provides comparative analyses in the form of pre-computed phylogenies of viral marker genes, rarefaction curves, and multivariate analyses based on K-mer signatures and BLAST-based comparison. While these comparative analyses can be useful for initial hypothesis generation, access to the input data is not provided thus limiting the utility of these analyses for more rigorous investigation. Ultimately, the most prudent approach is for the researcher to control and implement the entire workflow of comparative analyses from input data through to output statistics and graphs.
